# Linking Compositional and Functional Predictions to Decipher the Biogeochemical Significance in DFAA Turnover of Abundant Bacterioplankton Lineages in the North Sea

**DOI:** 10.3390/microorganisms5040068

**Published:** 2017-11-05

**Authors:** Bernd Wemheuer, Franziska Wemheuer, Dimitri Meier, Sara Billerbeck, Helge-Ansgar Giebel, Meinhard Simon, Christoph Scherber, Rolf Daniel

**Affiliations:** 1 Institute of Microbiology and Genetics, University of Göttingen, Grisebachstr. 8, D-37077 Göttingen, Germany; fwemheu@gwdg.de (F.W.); meier@microbial-ecology.net (D.M.); 2 Centre for Marine Bio-Innovation, School of Biological, Earth and Environmental Sciences, University of New South Wales, Sydney 2052, Australia; 3 Evolution and Ecology Research Centre, School of Biological, Earth and Environmental Sciences, University of New South Wales, Sydney 2052, Australia; 4 Institute for Chemistry and Biology of the Marine Environment (ICBM), University of Oldenburg, Carl-von-Ossietzky-Str. 9-11, D-26111 Oldenburg, Germany; sara.billerbeck@uni-oldenburg.de (S.B.); giebel@icbm.de (H.-A.G.); m.simon@icbm.de (M.S.); 5 Institute of Landscape Ecology, University of Muenster, Heisenbergstr. 2, D-48149 Muenster, Germany; christoph.scherber@uni-muenster.de

**Keywords:** microbial diversity, microbial ecology, structural modelling, functional predictions, marine microbiology

## Abstract

Deciphering the ecological traits of abundant marine bacteria is a major challenge in marine microbial ecology. In the current study, we linked compositional and functional predictions to elucidate such traits for abundant bacterioplankton lineages in the North Sea. For this purpose, we investigated entire and active bacterioplankton composition along a transect ranging from the German Bight to the northern North Sea by pyrotag sequencing of bacterial 16S rRNA genes and transcripts. Functional profiles were inferred from 16S rRNA data using Tax4Fun. Bacterioplankton communities were dominated by well-known marine lineages including clusters/genera that are affiliated with the *Roseobacter* group and the *Flavobacteria*. Variations in community composition and function were significantly explained by measured environmental and microbial properties. Turnover of dissolved free amino acids (DFAA) showed the strongest correlation to community composition and function. We applied multinomial models, which enabled us to identify bacterial lineages involved in DFAA turnover. For instance, the genus *Planktomarina* was more abundant at higher DFAA turnover rates, suggesting its vital role in amino acid degradation. Functional predictions further indicated that *Planktomarina* is involved in leucine and isoleucine degradation. Overall, our results provide novel insights into the biogeochemical significance of abundant bacterioplankton lineages in the North Sea.

## 1. Introduction

Bacteria are an integral component of marine ecosystems as key drivers of important nutrient cycles [1,2]. The application of novel sequencing techniques and meta-omics approaches has greatly advanced our understanding of bacterial communities and their ecological determinants in these ecosystems [3,4,5,6]. For example, analyses of entire and active bacterial communities along a salinity gradient in Delaware Bay by pyrotag sequencing revealed significant structural changes in surface water communities along the investigated gradient [7]. Recently, Zhang et al. [8] observed that the composition of entire and active bacterial communities along two transects in the South China Sea were strongly correlated with environmental factors, such as temperature or salinity. Despite the increasing number of studies on bacterioplankton communities, our knowledge of bacterial interactions with their environment is still limited [2,5,9]. Information on biogeochemical and functional traits of prominent marine bacterial lineages is often missing and is mostly based on genomic information that is retrieved from single isolates [10,11], which do not necessarily represent abundant marine lineages. This is exemplified by the unexpected discovery of respiratory nitrate reductases in members of the SAR11 clade [12], which can contribute to an anoxic lifestyle. Deciphering functional traits of prominent marine lineages is thus a major challenge in marine microbiology.

In recent years, bacterioplankton community dynamics have been studied extensively in the North Sea, and in particular its south-eastern part, the German Bight [13,14,15,16]. In two previous studies, the response of the bacterioplankton community towards a phytoplankton spring bloom in the German Bight was investigated by metagenomic and metatranscriptomic approaches [4,16]. Here, the abundance of several bacterial groups, such as the SAR92 clade and the *Roseobacter* clade-affiliated (RCA) cluster, were significantly affected by bloom presence. Other studies on bacterioplankton communities in the North Sea revealed that members of the *Roseobacter* group and the SAR11 clade were abundant and active in the North Sea [17,18,19]. However, detailed information on entire and active bacterioplankton community composition, especially in the northern North Sea, is still very limited. Moreover, we are still at the beginning to understand the impact of environmental changes on diversity, ecology, and functioning of abundant bacterioplankton lineages in the North Sea at fine scales.

Hence, the objective of this study was to identify the key drivers of entire and active bacterioplankton community composition and function in the North Sea. For this purpose, we investigated entire (DNA level) and putative metabolically active (RNA level) bacterioplankton community composition along a transect from the southern to the northern North Sea. The V3–V5 region of the 16S rRNA gene and its transcript was amplified by gene-specific PCR and RT-PCR, using environmental DNA and RNA as template, respectively. Subsequently, functional profiles were inferred from obtained 16S rRNA data using Tax4Fun [20]. We evaluated possible correlations between hydrographic, biogeochemical, and microbial properties to identify the main drivers of bacterioplankton community composition. Recently, Mock et al. [9] suggested that new interdisciplinary techniques, such as modelling approaches, are needed to bridge the gap between omics and earth system sciences. As we found a strong significant correlation between DFAA turnover rates and microbial community composition, we calculated multinomial models based on log-linear regression using measured DFAA turnover rates to identify bacterial groups related to the DFAA turnover. In addition, we used functional predictions to substantiate the notion that community function is associated with measured DFAA turnover rates. Linking structural and functional predictions enabled us to decipher potential ecological traits and the biogeochemical significance of abundant bacterioplankton lineages in the North Sea.

## 2. Materials and Methods

### 2.1. Study Site, Sampling and Sample Preparation

**Figure 1:**
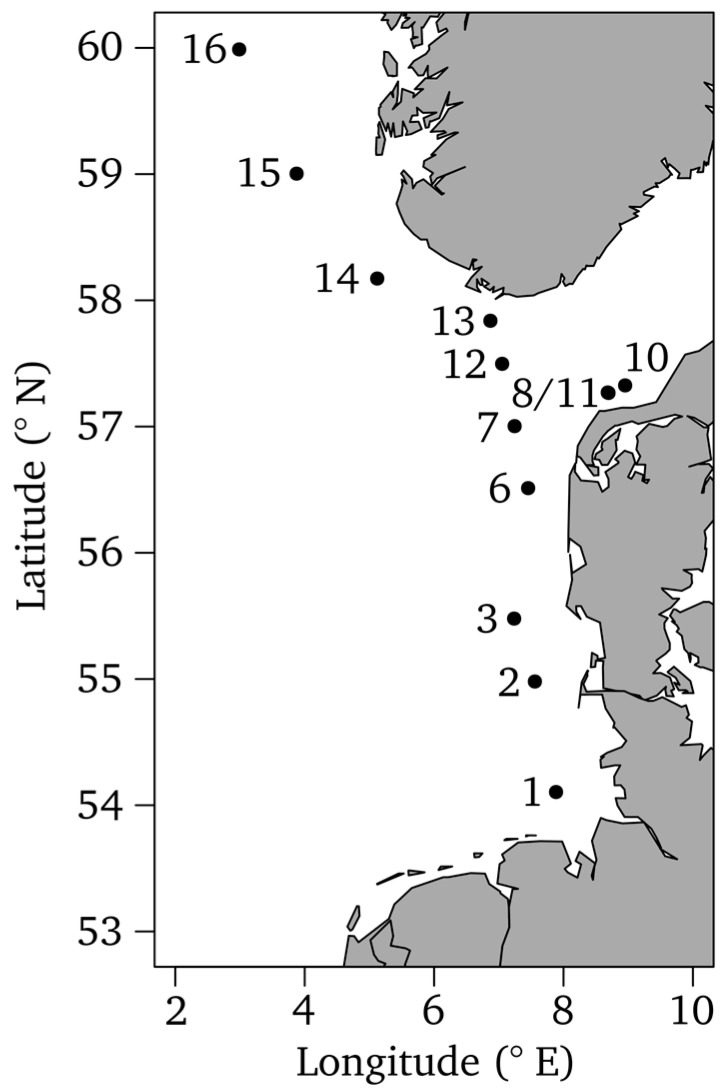
Map of the North Sea showing all samplings sites. Note that numbering follows ship stations. Stations 4, 5, and 9 are missing because no water samples were taken at these stations. Samples 8 and 11 were taken close to each other and are visualized as one station. The map was generated in [24] using the *maps* and *mapdata* packages [25,26].

The North Sea is a coastal sea with pronounced on-off shore and south-north gradients of inorganic nutrients, dissolved organic matter (DOM), phytoplankton biomass, and bacterioplankton growth [18,21,22]. During the last few decades, the North Sea, in particular the German Bight, underwent high nutrient loading and warming [21,23]. To investigate bacterioplankton community patterns in the North Sea, thirteen surface water samples were collected along an 800-km transect ranging from the German Bight at 54° N to 60° N west of Norway on board of the RV Heincke (Figure 1). 

Samples were taken using 5 L Niskin bottles mounted on a CTD rosette at 3 to 4 m depth from 15 to 20 July 2011 (Supplementary Table S1). Five samples (1, 2, 3, 6, and 7) were collected near the German and Danish coast, three samples (8, 10, and 11) in the Skagerrak, and five samples (12, 13, 14, 15, and 16) in the northern North Sea south and west of Norway (Figure 1). Note that numbering follows ship stations. Sampling and filtration were performed, as previously described [22]. Briefly, water of at least six Niskin bottles was pooled at each site for bacterioplankton community analyses. Obtained water samples were prefiltered through a 2.7-µm glass fiber filter (Whatman GF/D, GE Healthcare, Freiburg, Germany). Bacterioplankton was harvested from a prefiltered 10 L sample using a filter sandwich consisting of a 0.7-µm glass fiber filter (Whatman GF/F, GE Healthcare) and a 0.2-µm polycarbonate filter (Whatman Nuclepore, GE Healthcare). Filters were stored at −80 °C until further analysis.

Concentrations of chlorophyll *a* (chl *a*) and phaeopigments (phaeo) were determined spectrophotometrically after extraction in hot (75 °C) ethanol, according to Giebel et al. [18]. Biomass production of heterotrophic prokaryotes was determined by the incorporation of ^14^C-leucine, as described in Giebel et al. [18], and converted to carbon applying a leucine to carbon conversion factor of 3.05 kg C (mol leucine)^−1^ according to Simon and Azam [27]. Turnover rates of ^3^H-DFAA and ^3^H-glucose (glc) were determined as described for DFAA turnover in Giebel et al. [18]. Bacterioplankton cell numbers were determined by flow cytometry (BD AccuriTM C6, BD Biosciences, Heidelberg, Germany) using SybrGreen I staining and internal bead calibration as previously described [28]. For this purpose, water samples were preserved with glutaraldehyde (final concentration 1%), and stored at −20 °C until analysis.

### 2.2. Nucleic Acid Extraction and Sequencing

Total DNA and RNA were extracted from filters samples using acidic phenol and further purified as previously described [29]. DNA-free RNA was converted to cDNA according to Schneider et al. [29]. To assess community composition, the V3-V5 region of the bacterial 16S rRNA was amplified by PCR using universal primers according to Muyzer et al. [30]: 341f 5′-CCTACGGRAGGCAGCAG-3′ and 907r 5′-CCGTCAATTCMTTTGAGT-3 The PCR reaction was performed as described in Wemheuer et al. [31], with slight modifications. In brief, the PCR reaction (50 µL) contained 10 µL of 5-fold Phusion HF buffer (Thermo Fisher Scientific, Waltham, MA, USA), 200 µM of each of the four desoxynucleoside triphosphates, 1.5 mM MgCl_2_, 4 µM of each primer, 2.5% DMSO, 2 U of Phusion high fidelity hot start DNA polymerase (Thermo Fisher Scientific), and approximately 50 ng of DNA or 25 ng of cDNA as template. The following thermal cycling scheme was used: initial denaturation at 98 °C for 5 min, 25 cycles of denaturation at 98 °C for 45 s, annealing at 63 °C for 45 s, followed by extension at 72 °C for 30 s. The final extension was carried out at 72 °C for 5 min. Negative controls were performed using the reaction mixture without template. Obtained PCR products were purified by gel electrophoresis and quantified using the Quant-iT dsDNA HS assay kit and a Qubit fluorometer according to Wemheuer et al. [16]. The Göttingen Genomics Laboratory determined the sequences of the amplified PCR products using a Roche GS-FLX+ 454 pyrosequencer with Titanium chemistry (Roche, Mannheim, Germany).

### 2.3. Processing of 16S rRNA Data Sets

Obtained 16S rRNA data sets were processed, as described by Osterholz et al. [22]. Briefly, low quality reads (<25), sequences shorter than 250 bp, with more than three mismatches in the forward primer or homopolymers longer than 8 bp were removed prior to denoising with the Quantitative Insights Into Microbial Ecology (QIIME) software suite [32]. Remaining reverse primer sequences were truncated with cutadapt version 1.0 [33]. Chimeric sequences were removed using the UCHIME algorithm implemented in USEARCH version 7.0.190 [34] according to Wemheuer et al. [16]. Remaining sequences of all samples were clustered into operational taxonomic units (OTUs) at 97% genetic similarity [16]. To determine taxonomy, a consensus sequence for each OTU was generated and classified by BLAST alignment [35] against the Silva SSURef 119 NR database [36]. All of the non-bacterial OTUs were removed. Sequence statistics are provided as Supplementary Table S2. OTUs belonging to the *Roseobacter* OCT lineage were subsequently reclassified as previously described [22]. The final OTU table is provided as Supplementary Table S3. Accession numbers of reclassified sequences are listed in Supplementary Table S4. Alpha diversity indices were calculated with QIIME as described by Wemheuer et al. [4] (Supplementary Table S5). Functional profiles based on the obtained 16S rRNA data were predicted using Tax4Fun version 0.31 [20]. Tax4Fun transforms the SILVA-based OTU classification into a taxonomic profile of identical closely related genomes in the Kyoto Encyclopedia of Genes and Genomes (KEGG) database. Afterwards, these taxonomic profiles are converted into artificial metagenomes/metatranscriptomes by incorporating the functional data calculated from the genomes of each KEGG organism. Functional predictions are provided as Supplementary Tables S6 and S7. For this, OTU tables with unmodified Silva taxonomy were used. Predictions were performed with short read mode disabled. In addition, DNA-derived data was normalized for rRNA operon copy numbers.

### 2.4. Statistical Data Analysis

All statistical analyses were conducted in R version 3.2.2 [24] and the specific R packages listed below. Sample coverage was estimated using the Michaelis_Menten_Fit calculated in R. For this purpose, rarefaction curves were calculated using the *vegan* package [37]. The Michaelis_Menten_Fit was subsequently calculated from generated rarefaction curves using the *MM2* model within the *drc* package [38]. Rarefaction curves are provided as Supplementary Figure S1.

**Figure 2:**
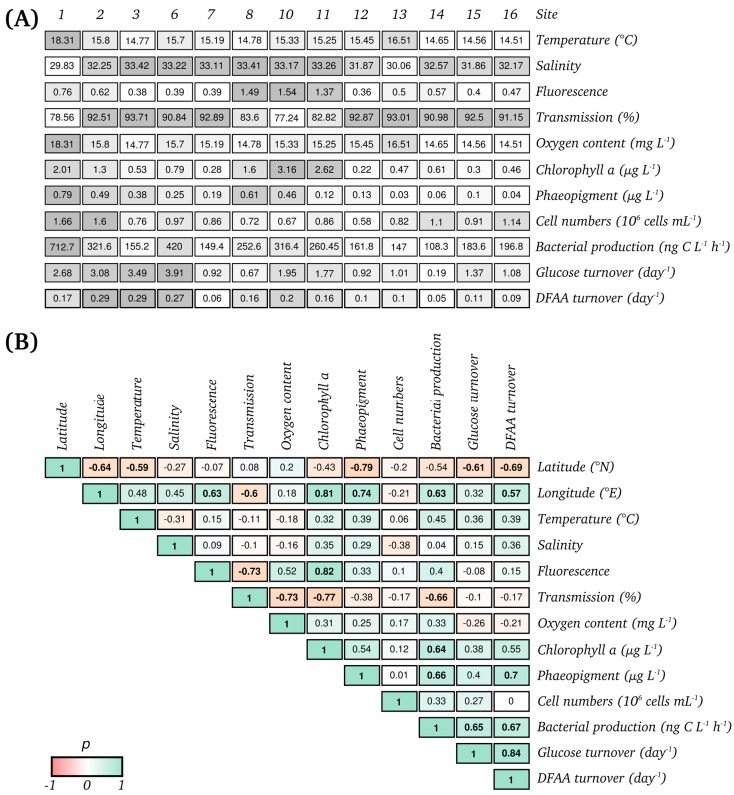
Environmental and microbial properties measured along the transect (**A**) and pairwise Spearman’s rank correlations between these properties (**B**). The shading in (**A**) accentuates the differences of environmental and microbial properties at each site (white to grey = lowest to highest value measured).

DNA and RNA derived data sets were analyzed separately to prevent pseudoreplication. All of the statistical tests performed in this study were considered significant at *p* ≤ 0.05. Correlations between geographical and environmental properties, as well as between DFAA turnover and predicted abundances of KEGG pathways and orthologs were determined by Spearman’s rank correlation (Figure 2B, Supplementary Tables S6 and S7). *p* values were adjusted for multiple comparisons according to Benjamini and Hochberg [39]. The impact of environmental and microbial properties on bacterial community composition was tested using permutational multivariate analysis of variance (PERMANOVA), based on weighted UniFrac dissimilarities in *vegan* [37]. In addition, Bray Curtis, unweighted UniFrac, variance-adjusted weighted UniFrac and Generalized UniFrac dissimilarities were tested, but displayed a lower environmental sensitivity due to lower average coefficients of determination (Supplementary Table S8). The distance matrix showing weighted UniFrac dissimilarities is provided as Supplementary Table S9. All of the UniFrac values were calculated using the *GUniFrac* package in R [40]. The phylogenetic tree necessary for the calculation of UniFrac distances was generated in QIIME [32], as previously described [16], with one modification: the tree was midpoint-rooted. Entire and active community composition were compared by the Mantel test in *vegan* [37], based on weighted UniFrac dissimilarities.

The impact of environmental and microbial properties on predicted functional profiles was tested using PERMANOVA based on Bray Curtis dissimilarities in *vegan* [37]. To verify that the observed correlation between DFAA and community function are not random, a total of 1000 randomized OTU tables were generated using the *permatfull* function of the *vegan* package [37] to build a null model. Afterwards, functional profiles were predicted from randomized OTU tables and were tested for correlations to DFAA turnover using PERMANOVA. More than 99% of these randomized predictions showed no or in comparison to the observed data a weaker correlation to DFAA turnover rates (data not shown).

Multinomial models were calculated based on log-linear regression models using the *multinom* function in the *nnet* package [41]. The response matrix used in the models contained all of the abundant bacterial marine groups (average abundance ≥ 1%). A total of 1000 randomized response matrices were generated using the *permatfull* function of the *vegan* package [37] to form a null model for the structural modelling approach. These matrices were subsequently linked to DFAA turnover. Structural predictions based on randomized OTU tables showed no obvious similarity to those derived from non-randomized OTU tables (See Supplementary Figure S2 as example). In addition, residual plots for multinomial models showed no obvious deviations from the assumptions of parametric statistics, such as an expected value of 0 and constant variance(See Supplementary Figure S3) (note that, for multi-category responses with moderate sample sizes, patterns in the residuals can be expected by chance alone).

### 2.5. Sequence Data Deposition

Sequence data was deposited in the Sequence Read Archive (SRA) of the National Center for Biotechnology Information (NCBI) under the accession number *SRA082674* as part of a study by Osterholz et al. [22], who investigated the DOM composition in the North Sea and its linkage to bacterioplankton community composition.

## 3. Results and Discussion

### 3.1. Biogeochemical and Microbial Characteristics

To investigate composition and functional traits of the bacterioplankton community in the North Sea, water samples were collected at thirteen stations along an 800-km transect, ranging from the German Bight to the west of Norway (Figure 1). Several hydrographic, biogeochemical, and microbial properties were measured along this transect (Figure 2A). Chl *a* and phaeo concentrations ranged from 1.3 to 3.2 µg L^−1^, and from 0.5 to 0.8 µg L^−1^, respectively, with the highest values in the German Bight (stations 1 and 2) and in the Skagerrak (stations 8, 10, 11). Phytoplankton blooms, dominated by dinoflagellates and diatoms, were present in these regions during the sampling campaign being responsible for the high chl *a* and phaeo concentrations. Bacterial biomass production varied between 108 and 713 ng C L^−1^ h^−1^ and exhibited the highest rates at the bloom stations and station 6 (Figure 2A). Bacterial cell numbers ranged from 0.6 and 1.7 10^6^ cells mL^−1^, with highest values at stations 1 and 2. 

Many environmental and microbial properties were significantly correlated with each other (Figure 2B). The concentration of phaeo, as well as the turnover of glc and DFAA, were negatively correlated to latitudinal change and decreased in northern samples. Fluorescence, biomass production, chl *a*, and phaeo concentrations were significantly linked to longitudinal change. Additionally, chl *a*, and phaeo were significantly correlated with bacterial production but not with bacterial cell numbers. Turnover rates of DFAA were significantly correlated with those of glucose.

### 3.2. Composition of Entire and Active Bacterioplankton Community in the North Sea

Entire and active bacterioplankton community composition were assessed by pyrotag sequencing of 16S rRNA amplicons that were generated from environmental DNA and RNA by PCR and reverse transcriptase (RT)-PCR, respectively. A total of 336,367 bacterial 16S rRNA sequences, with an average read length of 524 bp was retrieved recovering more than 80% of the bacterial richness based on coverage per sample estimated with the Michaelis_Menten_Fit (Supplementary Table S5). This is confirmed by the calculated rarefaction curves (Supplementary Figure S1). It should be noted that the results of the current study are only valid for the proportion of the community recovered by the surveying effort.

**Figure 3:**
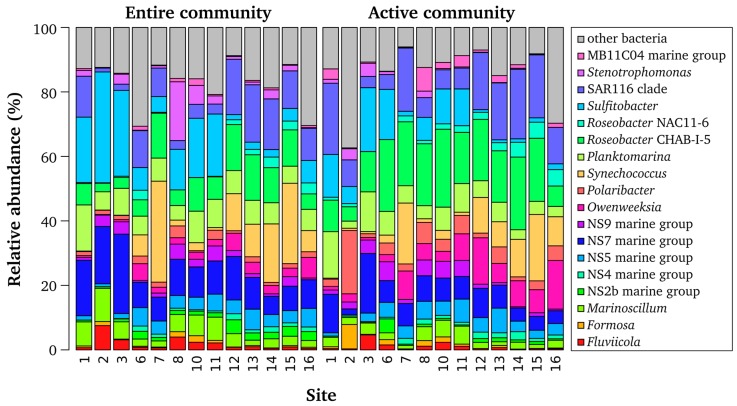
Observed composition of the entire and active bacterioplankton communities. Only genera and marine lineages with an average abundance of more than 1% are shown.

Consistent with previous work [4,13,16], bacterioplankton communities were dominated by 18 different lineages and genera belonging to the phyla *Proteobacteria*, *Bacteroidetes*, and *Cyanobacteria* (Figure 3). In a recent study investigating bacterioplankton communities in the North Sea during an algal bloom, *Proteobacteria* and *Bacteroidetes* were the most abundant bacterial phyla [16]. Similar results were observed in previous investigations of bacterial communities in the North Sea [13] and in surface seawater at 24 stations around the world [42]. Sequences affiliated to *Bacteroidetes* belonged to different genera and marine groups within the *Cytophagia-Flavobacteria,* such as the NS5 marine group. Sequences assigned to *Proteobacteria* mainly constituted lineages of *Alphaproteobacteria,* and belonged to the SAR116 clade and different clusters and genera of the *Roseobacter* group, including *Sulfitobacter*, NAC11-6, *Planktomarina temperata*-RCA, and CHAB-I-5. 

Although the first genomes for CHAB-I-5 [19,43] and *P. temperata*-RCA [15] became available recently, our knowledge on these particular members of the *Roseobacter* group, as well as their ecological role, is still limited. Interestingly, the SAR11 clade was observed in minor abundance, although this clade has been abundant in the surface waters of the North Sea [13,18,44] and other oceanic regions [45,46]. However, this finding is supported by the results of a previous study about bacterial communities in the North Sea during a phytoplankton bloom [4]. Here, SAR11 was less prominent when compared to other bacterial groups, such as *Roseobacter* RCA or SAR92. 

Observed differences in environmental/microbial properties, as well as in microbial community composition might be explained by the currents in the North Sea. The water depth of the southern part is less than 50 m and is subjected to strong tidal currents resulting in nutrient suspension from the sediment and loss of water stratification. In contrast, the northern part of the North Sea is deeper (up to 725 m) and strong tidal currents are not occurring. Overall, the results of the present study extend previous knowledge on bacterioplankton structure and diversity from the nutrient and plankton-rich southern North Sea to more nutrient depleted areas further north, as most previous studies investigated the southern region of the North Sea and the entire bacterial community only [6,13,16,47].

### 3.3. Entire and Active Bacterial Communities Displayed Differences in Richness and Community Composition

In this study, a higher genetic richness of the active bacterioplankton community was recorded compared to the entire bacterioplankton community (Supplementary Figure S1; Supplementary Table S5). This result is surprising, as we would assume that the active community is a fraction of the entire community. However, this observation supports a previous study of bacterial communities in the coastal Arctic Ocean where 16S rRNA clone libraries exhibited a lower diversity than the 16S rDNA clone libraries originating from the same samples [48]. A reason for the higher richness in our study might be that the abundance at RNA level is linked to cell abundance and to the number of rRNA transcripts per cell, which in turn corresponds to the protein synthesis rate [49]. Consequently, even rare species can be detected at RNA level as long as the low cell abundance is compensated by a high protein production. In addition, the number of 16S rRNA copies in a PCR reaction is higher when RNA is used as starting material as 16S rRNA transcripts constitute a major fraction of the total RNA [50]. 

The comparison of active and entire community composition revealed a divergent distribution pattern of several bacterial taxa (Figure 3, Supplementary Table S3). *Sulfitobacter* and *Synechococcus* were more prominent at the DNA level, whereas *Owenweeksia*, *Planktomarina*, and the *Roseobacter* cluster CHAB-I-5 were more abundant at RNA level. In addition, some low abundant phyla such as *Elusimicrobia, Fusobacteria*, *Gemmatimonadetes*, *Nitrospirae*, and *Spirochaetae* were observed at RNA level only (Supplementary Table S3). Thus, only a combined approach investigating the entire and active community will provide a more comprehensive picture of the bacterial community. A similar conclusion was reached in previous studies [8,48,51,52]. The analysis of active and entire bacterial community in the coastal Arctic revealed that some phylogenetic groups including the SAR11 clade were found at 16S rDNA level only [48]. In a study of bacterial communities in the South China Sea, OTUs belonging to *Cyanobacteria* and *Methylobacterium* were in low abundance in the DNA libraries, but were predominant in the RNA libraries, indicating that these bacteria are highly active [8]. In addition, high-abundant bacteria, including members of the SAR11 clade and *Rhodobacteriaceae*, were found only in minor abundance in the DNA libraries. The authors concluded that these bacteria might have a low activity. However, it should be noted that RNA abundance serves as an index but not as a measure of activity (as reviewed in [49]).

### 3.4. Bacterioplankton Community Composition and Function are Significantly Correlated to Environmental Properties

**Figure 4:**
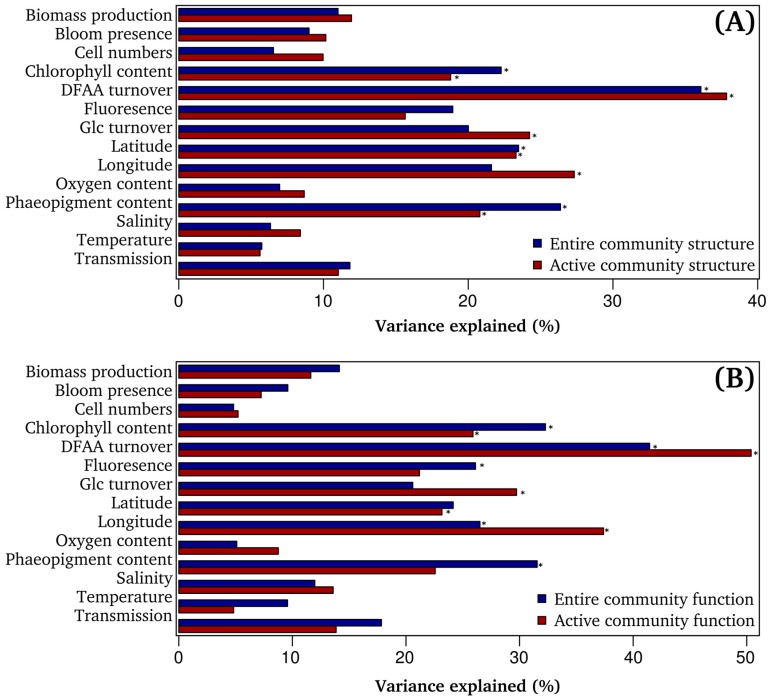
Correlation between environmental and microbial properties and observed bacterial community composition (**A**) as well as predicted functional profiles (**B**). Given is the coefficient of determination. Stars mark significant correlations. The correlation was tested by permutational multivariate analysis of variance (PERMANOVA) with 999 permutations.

Associations between hydrographic, biogeochemical, and microbial properties and bacterioplankton community composition (Figure 4A), as well as functional profiles (Figure 4B) were investigated using PERMANOVA. Several of these properties, such as latitude, turnover of DFAA, or concentration of chl *a* and phaeo were significantly correlated to bacterial community composition and function.

Despite the strong significant correlation (Mantel R = 0.64), the active bacterioplankton community composition displayed a slightly higher sensitivity to environmental properties than that of the entire community, as shown by the higher average coefficient of determination for active communities (Supplementary Table S8). A similar trend was reported by Zhang et al. [8] who investigated bacterioplankton communities in the South China Sea. They showed that the active heterotrophic bacterial community displayed tighter correlations to environmental properties than the entire community, indicating that entire and active fractions were controlled by different mechanisms.

We observed that concentrations of chl *a* and phaeo explained more of the variance of the composition in the entire compared to the active community. The stronger correlation of these bloom-related environmental properties with the entire bacterioplankton community suggests that a phytoplankton bloom event affects the entire community for a longer time period. In contrast, the active community represents the short-term adaptation to the current environmental conditions, with rapidly changing nutrient availabilities. This finding is also reflected by the fact that lineages of the active bacterioplankton exhibit different and more often closer correlations to distinct compounds of the DOM pool than those of the entire bacterioplankton community [22]. Our results further shed new light on previous findings by Teeling et al. [6,13], who detected a dynamic succession of different bacterioplankton lineages in response to a phytoplankton bloom. Given the differences in the PERMANOVA analysis that were obtained for both fractions (Figure 4A), our results corroborate the conclusion of Zhang et al. [8] that entire and active bacterial communities should be investigated simultaneously to gain better insights into the complex relation between bacterioplankton communities and environmental conditions. Our results, however, showed that it is most useful to include properties for bacterial growth and substrate uptake.

### 3.5. DFAA Turnover as Key Predictor of Bacterioplankton Community Composition

Interestingly, DFAA turnover rate explained more than 35% of the total variance in the dataset observed for community composition and function (Figure 4A,B). As DFAA turnover was significantly correlated with bacterial biomass production (Figure 2B), we suggest that DFAA are a main source of nitrogen and carbon for bacterial growth in the North Sea. Our findings support previous reports, which demonstrated that DFAA are essential substrates for the growth of (heterotrophic) bacterial communities in the German Wadden Sea [53], the Southern Ocean [54], the North Atlantic Ocean [55], the Central Arctic Ocean [56], and the Pacific Ocean [57]. Nonetheless, we expand existing knowledge as not only entire, but also active bacterioplankton community patterns were highly correlated with DFAA turnover.

**Figure 5:**
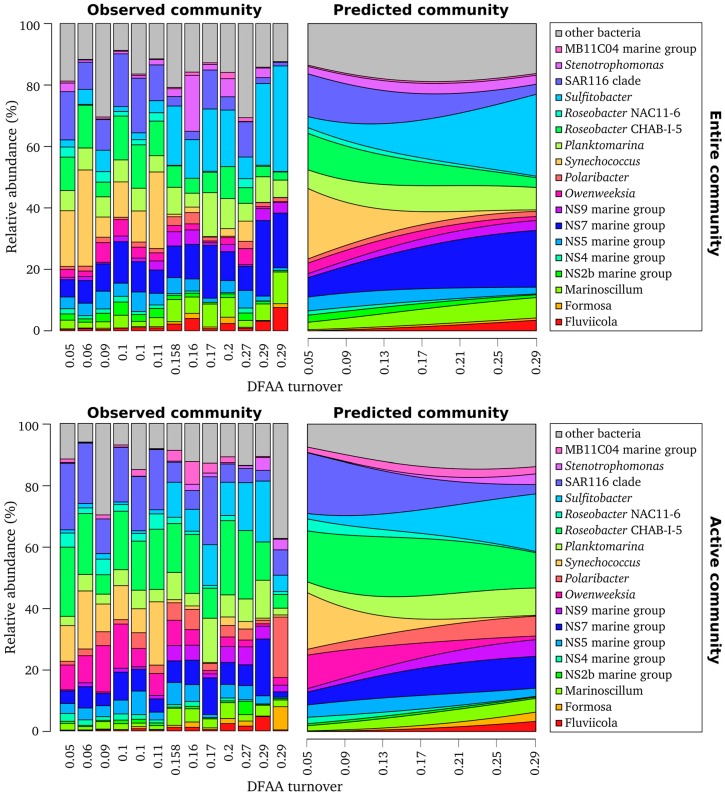
Observed and predicted composition of entire and active bacterioplankton communities in the North Sea. The measured dissolved free amino acids (DFAA) turnover rate is shown for the observed community. The stations are ordered from low to high DFAA turnover as follows: 14, 7, 16, 12, 13, 15, 11, 8, 1, 10, 6, 3, and 2. Compositional predictions were calculated based on observed community data using a multinomial log-linear model with DFAA turnover rates as sole explanatory variable. The range of the DFAA gradient used is equivalent to the DFAA turnover rates measured.

Recent advances in investigating marine microbial community dynamics have shown that the composition of marine bacterial communities follow predictable patterns and involves complex networks of interactions [5,58]. In a recent study, multinomial regression models via neural networks were successfully applied to investigate the structural responses of soil bacterial communities towards pH [59]. In the current study, we employed this approach to model the composition of the entire and active marine bacterial community composition as a function of DFAA turnover, the strongest predictor for community composition in our data set (Figure 5A).

The strong correlation of DFAA turnover might be explained by tight associations of several bacterial groups to the turnover of single amino acids. This is in line with an experimental study of Sarmento et al. [60] on phytoplankton species-specific release and bacterial uptake of DFAA. Here, the DFAA uptake was selective for certain members of heterotrophic bacterial communities, including the *Bacteroidetes* and *Roseobacter* group-related bacteria. Our modelling approach identified two genera of the *Roseobacter* group and a few lineages of *Bacteroidetes,* with an increasing abundance in the active community at rising DFAA turnover rates. For example, the genera *Planktomarina* (formerly known as the RCA cluster) and *Sulfitobacter* as well as the NS7 marine group were more abundant at high DFAA turnover rates (Figure 5A), indicating that these genera are involved in the DFAA degradation. Our findings support the results of Giebel et al. [18] who found that important bacterial lineages in the North Sea, such as the RCA cluster and the SAR11 clade, were positively correlated with DFAA turnover. Hence, our results from a field study and modelling approach supports the experimental study by Sarmiento et al. [60].

Other lineages, most prominently the SAR116 clade and the photoautotrophic *Synechococcus*, but also the NAC11-6 lineage of the *Roseobacter* group, were negatively correlated to DFAA turnover in our modelling approach. These results indicate that these groups are either involved in DFAA synthesis or that they are independent of exogenous DFAA supply. Interestingly, a similar pattern was observed for the *Roseobacter* CHAB-I-5 cluster, but at DNA level only. Its abundance in the active community was highest at intermediate turnover levels, indicating that this cluster is involved in both, DFAA synthesis and degradation.

### 3.6. Community Function is Significantly Linked to Measured DFAA Turnover Rates

**Figure 6:**
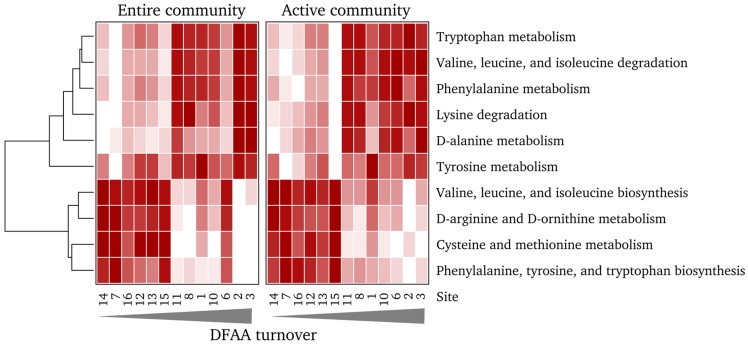
Abundance of KEGG pathways putatively involved in amino acid metabolism and significantly linked to DFAA turnover. White: low relative abundance; red: high relative abundance. Numbers correspond to sampling stations.

To assess how community functions are associated to DFAA turnover rates, we further linked DFAA turnover rates and functional predictions focusing on pathways related to amino acid metabolism (Figure 6; Supplementary Table S6). Moreover, we compared functional profiles that are derived from randomized OTU tables with profiles that were predicted from observed community data. Functional profiles inferred from randomized OTU tables showed very low or no correlation to DFAA turnover (observed: DNA/RNA R^2^ = 0.402/0.489; randomized: DNA/RNA 0.0788±0.067/0.115±0.076; with 1000 iterations).

We identified several pathways that were significantly linked to measured DFAA turnover rates (Supplementary Table S6). Predicted abundances of pathways involved in the degradation of non-polar and aromatic amino acids were higher in samples exhibiting high DFAA turnover rates, whereas the opposite was found for the pathways involved in the biosynthesis of these amino acids and in the metabolism of cysteine and methionine. These findings suggest that the non-polar and aromatic amino acids are primarily degraded when DFAA turnover rates are high, whereas their biosynthesis and the metabolism of the two sulfur-containing amino acids is enhanced when turnover is low. This approach thus appears to provide a refined insight into how the different amino acids are metabolized in distinctly different ways. As mentioned earlier, the abundance of two marine lineages, the genera *Planktomarina* and *Sulfitobacter*, as well as the NS7 marine group, strongly increased with rising DFAA turnover rates (Figure 5A). This indicates that these groups are major players in the degradation of non-polar and aromatic amino acids in the North Sea. The biosynthesis of these amino acids might be attributed to members of the SAR116 clade, *Synechococcus*, and the genus *Owenweeksia*, which were more abundant in samples displaying low DFAA turnover rates. The CHAB-I-5 cluster appears to be able to cope in its properties of amino acid degradation and biosynthesis with rather variable DFAA turnover rates.

### 3.7. Study Limitations

A total of thirteen surface water samples were taken during the research cruise presented here. Although these samples were mixed samples consisting of at least six Niskin bottles (5L water per bottle), further cruises should include more biological replicates/stations. Moreover, a long-term study covering different seasons is merited as observed results might change over time. In addition, the strong correlation of DFAA turnover rate and microbial community composition as well as function might only be valid for the North Sea because microbial communities in other pelagial ecosystems encounter different nutrient availabilities and other prevailing environmental conditions. Therefore, more studies investigating bacterioplankton communities in other pelagial systems are needed to validate the results of the current study. Further cruises should also measure the DFAA composition as well as phosphorous and nitrogen content in the water samples to provide additional data for the compositional modelling approach.

## 4. Conclusions

The current understanding of function and ecology of abundant bacterioplankton lineages in pelagic marine systems is still very limited when considering the few cultured or genomically explored members of these lineages. Here, we investigated the ecological traits of abundant bacterioplankton lineages in the North Sea by linking compositional and functional predictions to environmental and microbial properties. Turnover rates of DFAA showed the highest correlation to observed community composition and function, and, thus, were used to model bacterial community composition. This model identified prominent bacterial lineages, such as the SAR116 clade probably involved in the DFAA turnover. Functional profiles inferred from 16S rRNA data using Tax4Fun provided reliable insights into community functioning, as indicated by the strong association of functions that were related to biosynthesis and degradation of distinct amino acids and measured DFAA turnover rates. This study highlights the importance of examining both bacterial 16S rRNA genes and 16S rRNA transcripts to obtain the full picture of a bacterial community and its dynamics as richness and community composition differed between the entire and active bacterial communities in the North Sea. Moreover, we demonstrated the power of combining compositional and functional predictions to decipher ecological traits of abundant bacteria in the North Sea. Nonetheless, empirical evidence is needed to confirm the results of the current study.

## Supplementary Materials

The following are available online at www.mdpi.com/2076-2607/5/4/68/s1.
