# Supplementary material for: Linking Compositional and Functional Predictions to Decipher the Biogeochemical Significance in DFAA Turnover of Abundant Bacterioplankton Lineages in the North Sea
Source: Microorganisms. 2017 Nov 5;5(4):68. doi: 10.3390/microorganisms5040068 (PMC5748577; doi:10.3390/microorganisms5040068)

Residuals from multinomial model DNA vs. DFAA turnover

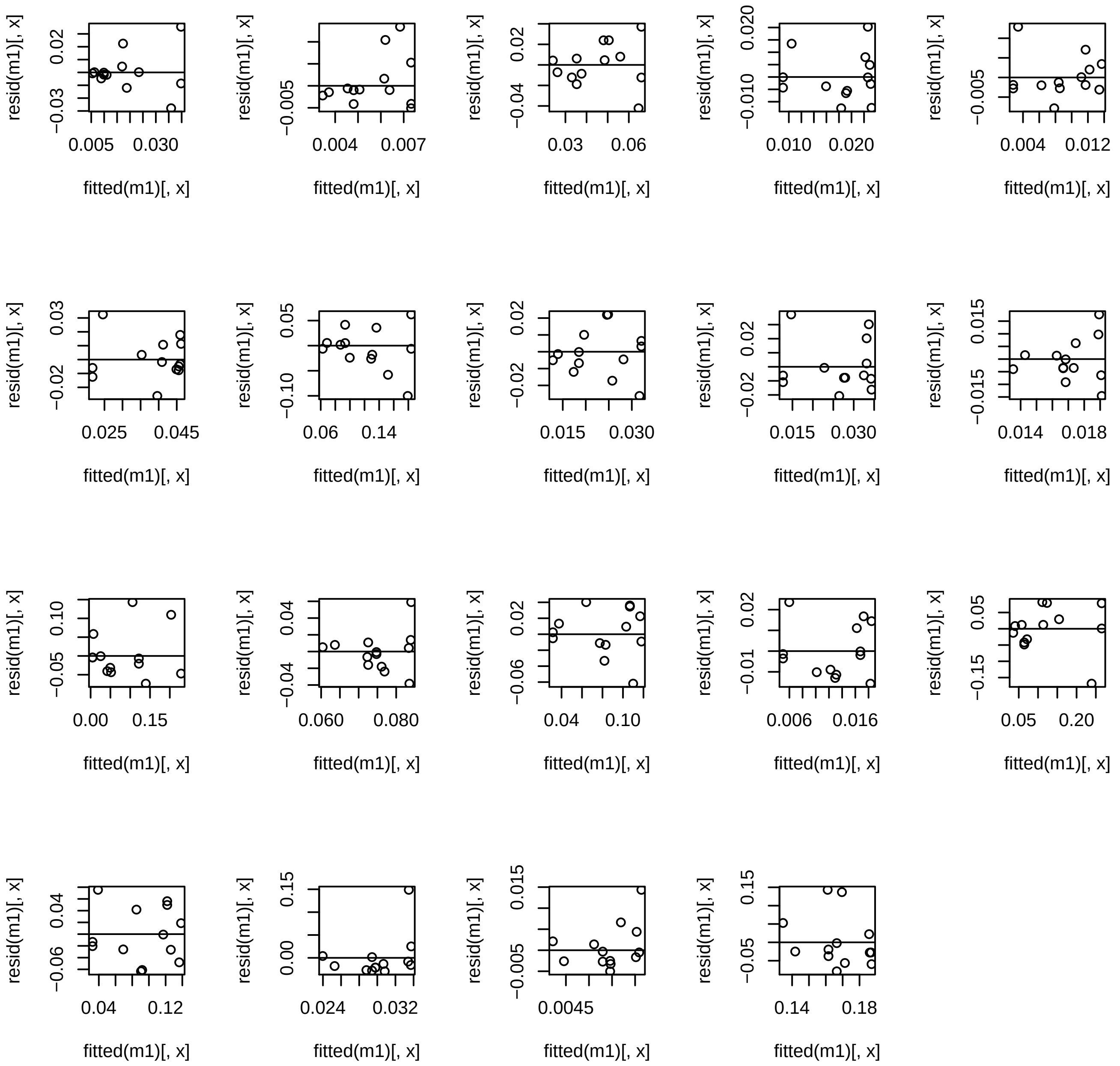

Residuals from multinomial model RNA vs. DFAA turnover

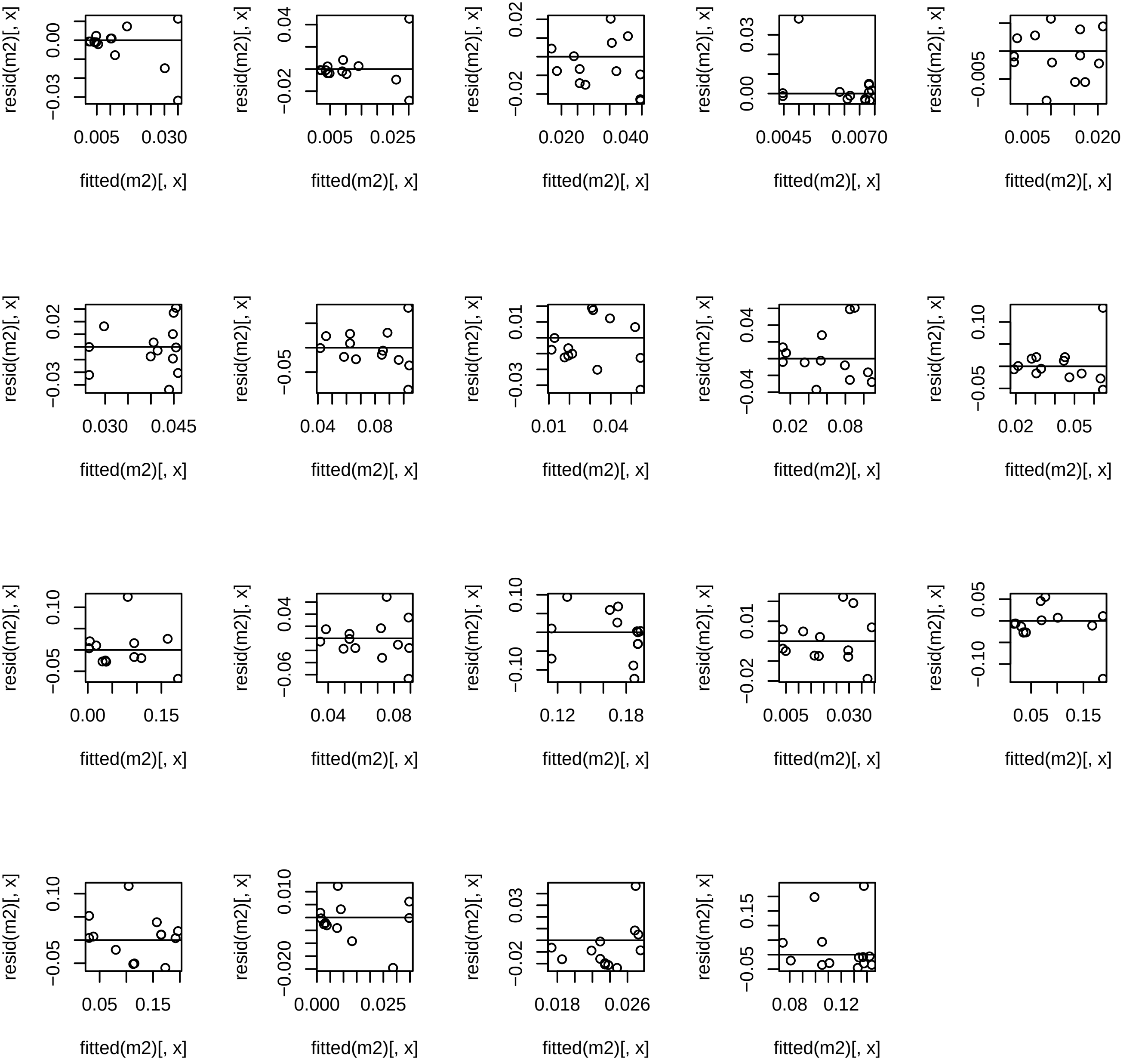

Supplement: Supplementary file 1 [file microorganisms-05-00068-s001.zip › FigureS3.pdf]
